# Soluble ST2 as a Potential Biomarker for Abdominal Aortic Aneurysms—A Single-Center Retrospective Cohort Study

**DOI:** 10.3390/ijms23179598

**Published:** 2022-08-24

**Authors:** Johannes Klopf, Svitlana Demyanets, Mira Brekalo, Wolf Eilenberg, Johann Wojta, Christoph Neumayer, Christine Brostjan, Stefan Stojkovic

**Affiliations:** 1 Department of General Surgery, Division of Vascular Surgery, University Hospital Vienna, Medical University of Vienna, 1090 Vienna, Austria; 2 Department of Laboratory Medicine, University Hospital Vienna, Medical University of Vienna, 1090 Vienna, Austria; 3 Department of Internal Medicine II, Division of Cardiology, University Hospital Vienna, Medical University of Vienna, 1090 Vienna, Austria; 4 Ludwig Boltzmann Institute for Cardiovascular Research, 1090 Vienna, Austria; 5 Core Facilities, Medical University of Vienna, 1090 Vienna, Austria

**Keywords:** abdominal aortic aneurysm (AAA), soluble suppression of tumorigenesis 2 (sST2), interleukin 33 (IL-33), diagnosis, growth prediction

## Abstract

The maximal aortic diameter is the only clinically applied predictor of abdominal aortic aneurysm (AAA) progression and indicator for surgical repair. Circulating biomarkers resulting from AAA pathogenesis are attractive candidates for the diagnosis and prognosis of aneurysmal disease. Due to the reported role of interleukin 33 in AAA development, we investigated the corresponding circulating receptor molecules of soluble suppression of tumorigenesis 2 (sST2) in AAA patients regarding their marker potential in diagnosis and prognosis. We conducted a single-center retrospective cohort study in a diagnostic setting, measuring the circulating serum sST2 protein levels of 47 AAA patients under surveillance, matched with 25 peripheral artery disease (PAD) patients and 25 healthy controls. In a prognostic setting, we analyzed the longitudinal monitoring data of 50 monitored AAA patients. Slow versus fast AAA progression was defined as a <2 or ≥2 mm increase in AAA diameter over 6 months and a <4 or ≥4 mm increase over 12 months. Additionally, the association of circulating serum sST2 and AAA growth was investigated using a specifically tailored log-linear mixed model. Serum sST2 concentrations were significantly increased in AAA patients compared with healthy individuals: the median of AAA patient cohort was 112.72 ng/mL (*p* = 0.025) and that of AAA patient cohort 2 was 14.32 ng/mL (*p* = 0.039) versus healthy controls (8.82 ng/mL). Likewise, PAD patients showed significantly elevated sST2 protein levels compared with healthy controls (the median was 12.10 ng/mL; *p* = 0.048) but similar concentrations to AAA patients. Additionally, sST2 protein levels were found to be unsuited to identifying fast AAA progression over short-term periods of 6 or 12 months, which was confirmed by a log-linear mixed model. In conclusion, the significantly elevated protein levels of sST2 detected in patients with vascular disease may be useful in the early diagnosis of AAA but cannot distinguish between AAA and PAD or predict AAA progression.

## 1. Introduction

An abdominal aortic aneurysm (AAA) is a focal, full-thickness dilatation of the abdominal aorta and is diagnosed when the maximal aortic diameter exceeds 30 mm [1,2]. The natural course of AAA is characterized by a typically asymptomatic disease progression with indefinite growth and risk of rupture, with a 50–90% mortality rate if left undiagnosed or untreated [3]. Advanced age, male sex, smoking, a positive family history and the presence of other cardiovascular diseases such as hypertension, hyperlipidemia, ischemic heart disease or peripheral artery disease (PAD) are risk factors for AAA [4]. Most preclinical and clinical studies have indicated chronic aortic inflammation as being crucial in AAA pathogenesis. Multifactorial inflammatory processes such as immune cell infiltration, extracellular matrix degradation, the release of proteases and cytokines, and generation of reactive oxygen species lead to aortic vessel wall weakening and destruction [5,6,7,8]. To date, the maximal aortic diameter is the only clinically approved predictor of AAA progression and indicator for surgery [1,2,9]. However, aneurysm diameter alone does not always accurately represent the individual risk of fast disease progression or rupture. Additional prognostic parameters are greatly warranted. Considering AAA etiology, pro-inflammatory markers such as interleukin (IL)-33 and its decoy receptor, soluble suppression of tumorigenesis 2 (sST2), may potentially reflect the AAA disease state and thus seem promising parameters for risk stratification [10].

The IL-33/sST2 pathway has been implicated in multiple acute and chronic inflammatory pathologies [11]. It modulates angiogenesis and extracellular matrix degradation by regulating the plasminogen activation system [12]. Monocytes and macrophages constitutively express the ST2 receptor, and IL-33 modifies macrophage polarization, causing a pro- or anti-inflammatory response depending on the disease and the pathomechanistic model [13,14,15,16]. Several primary cell and organ sources of sST2, including the arterial endothelial cells, have been reported. The vascular endothelium perceives hemodynamic and inflammatory conditions, and secretes sST2 into the circulation. Soluble ST2 reflects the activation of the IL-33/ST2 pathway and constitutes an autologous feedback loop for downregulation, i.e., multifunctional IL-33 exerts its advantageous protective effects by binding to the membrane-bound ST2 receptor ST2L, which can be competitively blocked by soluble ST2 molecules [10,17,18,19]. Otherwise, soluble ST2 may function as a decoy receptor for the pro-inflammatory activity of IL-33 within the inflamed aneurysmal environment, which is released to alleviate the consequences of excessive IL-33 exposure [20]. A recent preclinical study suggested the critical role of the IL-33/sST2 pathway in suppressing AAA pathogenesis [21]. Increased IL-33 expression was demonstrated in an experimental murine AAA model, where it was predominately localized in the aortic fibroblasts. Overexpression of IL-33 decreased AAA size as well as the infiltration of T-cells and macrophages, with a shift to M2 macrophage polarization [21]. Since soluble ST2 receptor negatively regulates IL-33 signaling, it would be expected to promote aneurysm formation and rise upon AAA development [22,23]. Soluble ST2 is an established prognostic biomarker for acute and chronic heart failure, as well as for acute aortic dissection [24,25]. To date, there are no studies investigating the biomarker potential of sST2 in human AAA. Given the potent role of the IL-33/sST2 pathway in murine models of AAA, we hypothesized that circulating sST2 blood levels might be elevated in AAA patients, reflect he advanced AAA disease state and predict the rate of AAA progression [21]. Here, we aimed to investigate the diagnostic and prognostic value of sST2 for AAA disease state and aneurysm progression.

## 2. Results

### 2.1. Patient and Control Collectives

Forty-seven AAA patients, divided into two patient cohorts, alongside 25 healthy controls and 25 PAD patients were included in the retrospective diagnostic analysis. The groups were matched for sex and age (±2 years) and as well as possible for smoking habit. The AAA and PAD cohorts were additionally matched for previous cardiovascular events (such as myocardial infarction, coronary heart disease and stroke). This detailed one-to-one matching (with paired statistical tests) was conducted for two AAA cohorts, allowing an assessment of reproducibility. The patients’ and controls’ demographics are listed in Table 1. Overall, the groups were comparable in terms of age, body mass index, smoking behavior and blood pressure, with a few exceptions where significant differences were recorded despite a very close data range such as the age distribution of AAA cohort 1 versus healthy controls, with median (IQR) values of 67.6 (11) and 67.3 (11) years, respectively (*p* = 0.009). Patients with abdominal aortic aneurysm were divided into AAA patient cohort 1 (*n* = 25) and AAA patient cohort 2 (*n* = 22), and did not show significant differences in AAA maximal diameter, aortic segment volume and associated intraluminal thrombus (ILT) morphology, as well as AAA family history. Most study participants were men, with 92–95.5% in each study cohort. No significant differences with regards to systolic and diastolic blood pressure were observed among the groups, except for an increased mean systolic blood pressure in PAD patients compared with healthy controls. More patients with AAA and PAD were affected with hyperlipidemia and coronary heart disease compared with healthy individuals. In addition, patients in AAA cohort 1 and PAD patients showed a significantly higher prevalence of hypertension and diabetes mellitus type 2 compared with healthy controls. Other comorbidities and cardiovascular events such as myocardial infarction and stroke showed no significant variability among the healthy control, AAA and PAD collectives. On the contrary, the diseased groups differed significantly, with an almost 1.5-fold to sixfold higher frequency of antiplatelet, antihypertensive and lipid-lowering therapy compared with healthy controls. Further details of the patient population characteristics and concomitant medication are listed in Table 1.

In a prognostic study design, 50 AAA patients without an indication for surgical repair were followed at 6-month intervals (median: 5.98 months ± 0.46 IQR) for observations of AAA expansion during a period of 1 year. This reflects a total of 100 monitoring periods. The AAA patient demographics of this prognostic study cohort are listed in Table 2. Seven women (14.0%) and 43 men (86.0%) with a median age of 71.4 years at study entry were included. The median body mass index was within the range of overweight at 27.6 kg/m^2^. This AAA patient group reached a median maximal aneurysm diameter of 43.7 mm with a median aortic segment volume of 68.2 cm^3^. Furthermore, a median maximal ILT thickness of 12.3 mm and median ILT volume of 24.5 cm^3^ were recorded. A median of 40 smoking pack-years reflects the significant prevalence of smoking among AAA patients. The most frequent comorbidities of the prognostic AAA patient collective were hypertension and hyperlipidemia (90% and 84%, respectively). Antihypertensive agents (88%), antiplatelet medication (92%) and lipid-lowering agents (94%) were the drugs prescribed most often in this AAA patient population. Table 2 provides further details on the patients’ demographic characteristics and concomitant medication. 

### 2.2. Soluble ST2 Is Increased in AAA and PAD

**Figure 1:**
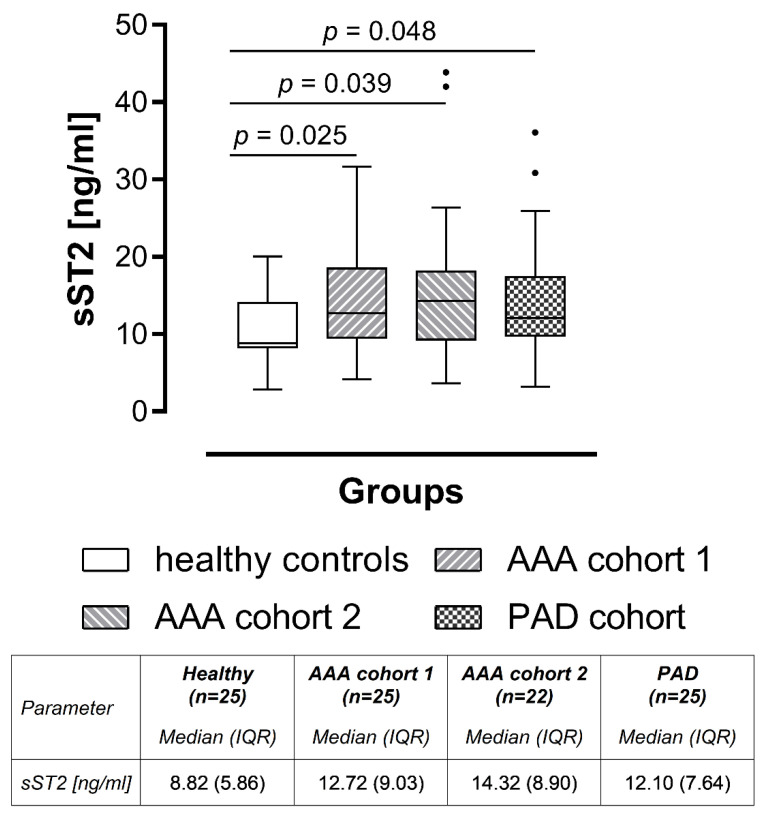
Comparison of circulating sST2 concentrations between AAA patients and control groups. For the diagnostic study, circulating sST2 concentrations were measured in serum samples from 25 healthy controls, matched one-to-one (age, sex, best possible in smoking habit) with 25 AAA cohort 1 patients, 22 AAA cohort 2 patients and 25 PAD patients (additionally matched for previous cardiovascular disease). Statistical analysis was performed by using a Wilcoxon signed rank test, and the boxplot illustrates the sST2 values according to Tukey’s style. Abbreviations: sST2, soluble suppression of tumorigenesis 2; AAA, abdominal aortic aneurysm; PAD, peripheral artery disease; *n*, number of individuals; IQR, interquartile range.

**Figure 2:**
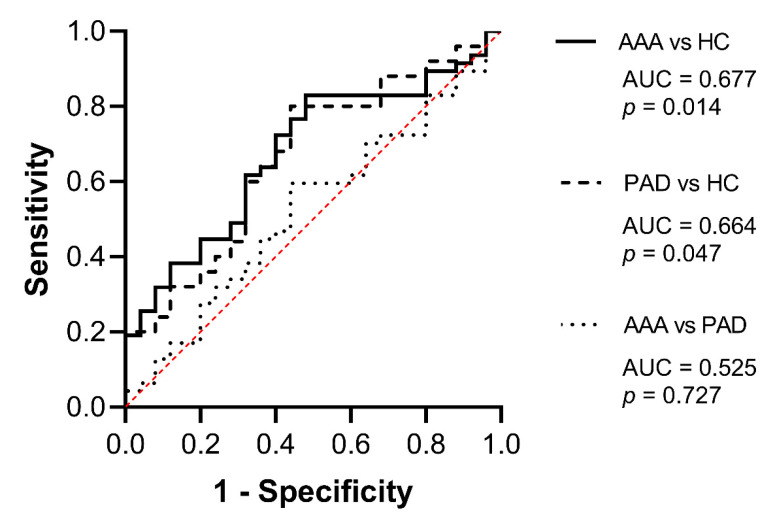
Diagnostic marker potential of sST2. The diagnostic marker value of sST2 was further evaluated by receiver operating characteristic (ROC) analysis and the area under the ROC curve (AUC). Abbreviations: AAA, combined AAA patient cohorts 1 and 2; HC, healthy controls, PAD, peripheral artery disease patient cohort.

The explorative blood parameter sST2 showed a significant elevation in both AAA patient cohorts compared with the healthy control collective (Figure 1). We observed a 1.4-fold increase in median sST2 protein concentrations in AAA patient cohort 1 (12.72 vs. 8.82 ng/mL, *p* = 0.025) and a 1.6-fold sST2 protein level elevation in AAA patient cohort 2 (14.32 vs. 8.82 ng/mL, *p* = 0.039) compared with the healthy control group, respectively. AAA patient cohorts 1 and 2 did not differ significantly (12.72 vs. 14.32 ng/mL, *p* = 0.661). Additionally, we evaluated the circulating sST2 concentrations of healthy individuals and AAA patients in relation to the sST2 levels of PAD patients (without aneurysms) to address the question whether sST2 is a biomarker which can distinguish AAA from other types of vascular disease. The circulating sST2 concentrations in AAA patients and control subjects (healthy controls and PAD) are illustrated in Figure 1. Patients with PAD showed significantly higher values of sST2 compared with healthy controls (12.10 vs. 8.82 ng/mL, *p* = 0.048). No significant differences were found between AAA patient cohort 1 (12.72 vs. 12.10 ng/mL, *p* = 0.946) or AAA patient cohort 2 (14.32 vs. 12.10 ng/mL, *p* = 0.592) and PAD patients, respectively. Receiver operating characteristic (ROC) analysis and the area under the ROC curve (AUC), as illustrated in Figure 2, were used to further assess the diagnostic marker value of sST2. Thus, circulating sST2 protein levels significantly discriminated between AAA patients (combined AAA patient cohorts 1 and 2) and healthy individuals (AUC = 0.677, *p* = 0.014). Likewise, PAD patients could be identified by elevated sST2 levels compared with healthy controls (AUC = 0.664, *p* = 0.047). In line with our observation that sST2 concentrations are increased in both types of vascular disease, serum protein levels of sST2 could not discriminate between AAA and PAD patients (AUC = 0.525, *p* = 0.727).

### 2.3. Soluble ST2 Levels Are Only Weakly Associated with AAA Progression

**Figure 3:**
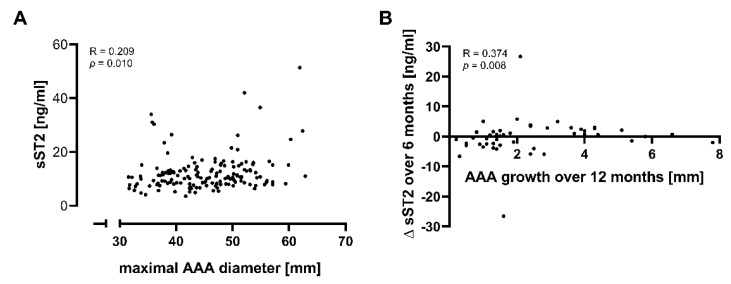
Significant correlations of sST2 concentration and AAA diameter, and their change over time. (**A**) Association between serum sST2 concentrations and maximal AAA diameter. For the prognostic study, circulating sST2 concentrations were longitudinally measured in serum samples from 50 AAA patients at 6-month intervals, resulting in a total of 150 monitoring datapoints. A correlation analysis of sST2 levels and maximal AAA diameter was based on the Spearman correlation coefficient and illustrated with a scatterplot. (**B**) Association between parameter changes. Spearman’s correlation between the change (∆) in sST2 concentrations over 6 months and the increase in maximal AAA diameter (AAA growth) over 12 months, illustrated by a scatterplot. Abbreviations: sST2, soluble suppression of tumorigenesis 2; AAA, abdominal aortic aneurysm.

Next, we monitored circulating sST2 and aneurysm growth in 50 AAA patients over the timeframe of 1 year, covering three time points at baseline, 6 months and 12 months. A weak but significant correlation of circulating sST2 values and maximal aneurysm diameter was found (R = 0.209, *p* = 0.010), as illustrated in Figure 3A. We further evaluated the biomarker potential of serum sST2 to predict AAA growth over the next 6 or 12 months. However, protein serum levels of sST2 did not differ significantly between patients with slow and rapid AAA progression, defined as a <2 or ≥2 mm increase in AAA diameter over 6 months, with median values of 11.01 (IQR = 5.99) vs. 10.16 (IQR = 5.12) ng/mL (*p* = 0.444), respectively. Likewise, there was no prognostic value for sST2 in predicting fast (≥4 mm) AAA progression over a 12-month period (median 10.93 (IQR = 5.83) vs. 10.62 (IQR = 5.03) ng/mL, *p* = 0.511). Furthermore, no significant correlations between starting protein levels of sST2 and an increase in maximum AAA diameter over the following 6 or 12 months were observed (R = −0.013, *p* = 0.898 and R = −0.086, *p* = 0.553, respectively). Interestingly, we found a significant correlation between the change in sST2 protein concentrations over the first 6 months and the increase in maximal AAA diameter over 12 months (R = 0.374, *p* = 0.008), as shown in Figure 3B. Additionally, we applied a log-linear mixed model accounting for multiple measurements in AAA patients over time to evaluate the biomarker potential of sST2 for predicting AAA progression. A non-significant interaction (*p* = 0.367) between serum sST2 levels and time interval affirmatively indicated the lack of prognostic power for AAA progression.

## 3. Discussion

This study focused on an evaluation of sST2 as a member of the IL-1 receptor family and its association with AAA disease state and progression. We aimed to establish its possible diagnostic and prognostic marker potential by analyzing circulating protein levels of sST2 in AAA patients. We showed that AAA patients have elevated circulating sST2 concentrations compared with healthy controls. Furthermore, sST2 was shown to be elevated in patients with PAD (without aneurysms) but could not significantly discriminate between AAA and PAD patients. Additionally, serum sST2 concentrations were found to be unsuited to identify and predict fast AAA progression over short-term periods of 6 or 12 months. Of note, two AAA cohorts differing in the comorbidities of hypertension, diabetes mellitus type 2 and chronic obstructive pulmonary disease gave comparable results (one-to-one matched for sex, age (±2 years), the presence of clinical cardiovascular disease (previous myocardial infarction, angina pectoris, peripheral vascular disease or cerebrovascular disease) and best possible in smoking habit)).

A recent preclinical study revealed that mice are protected from AAA formation by enhancement of membrane-bound ST2-dependant immunosuppressive T-cell expansion [21]. Summarizing the manifold aspects of aneurysm pathophysiology, AAA is mainly considered a chronic inflammatory disease, where immunological mediators release detrimental constituents and substances at the aneurysm site which contribute to the destruction of the aortic wall [6,26]. However, very little is known about sST2’s origin measured in the circulation, or its expression patterns, mechanisms of release and function in the aorta, particularly in humans. The IL-33/sST2 system was detected in the aortas and cultured vascular smooth muscle cells of Wistar rats. The study indicated that a high-fat diet increased aortic sST2, with deleterious effects on vascular remodeling [27]. This association might also apply to AAA and PAD patients, who often present with hyperlipidemia and an increased body mass index. One can speculate that sST2 is produced during AAA formation as a result of aortic dilation or aneurysm-related pathophysiological inflammatory and tissue-destructive mechanisms [17,18]. Additionally, it is highly relevant, but unknown, if sST2 is primarily released by resident aortic cells and/or by inflammatory cells that have migrated to the aneurysm site [28]. As a multifunctional cytokine in vascular disease, IL-33 exerts its advantageous protective effects by binding to the membrane-bound ST2 receptor ST2L, which can be competitively blocked by soluble ST2 molecules [10,19]. Alternatively, sST2 may function as a decoy receptor for the pro-inflammatory activity of IL-33 within the inflamed aneurysmal environment, which is released to alleviate the consequences of excessive IL-33 exposure. Consequently, an appropriate amount of sST2 can prevent uncontrolled aortic inflammation, as present in healthy individuals [20].

Soluble ST2 has previously been shown to be an effective biomarker of cardiac diseases and their progression; in particular, sST2 is an established prognostic biomarker and predictor of mortality for myocardial infarction and heart failure [10,29,30,31,32]. Recently, a study reported on the high diagnostic accuracy of sST2 in detecting acute aortic dissection [25]. The fact that sST2 is known to be elevated in cardiovascular diseases other than AAA may explain why serum sST2 did not exhibit discriminatory power between AAA and PAD [10,33]. Additionally, baseline sST2 protein levels did not correlate with subsequent AAA growth or differ significantly between slow and fast AAA progressors. Unexpectedly, we observed a significant correlation between the change in sST2 protein concentrations over the first 6 months and the increase in maximal AAA diameter over 12 months. This may indicate that a rise in sST2 levels within the first 6 months of monitoring reflects increased inflammatory AAA activity and may hence predict a faster rate of AAA expansion over the entire monitoring period of 12 months.

Finally, there are several study limitations, which should be addressed. The data for this study were collected retrospectively and the sample size of our study, although based on carefully matched collectives, has certainly limited the assessment of the diagnostic and prognostic marker potential. The study was explorative in design, so no adjustments for a multiplicity of errors were used, increasing the possibility of experiencing a type I error. We used the human ST2/IL-1 R4 DuoSet ELISA kit (R&D Systems, Minneapolis, MN, USA). For sST2 protein measurements, two other assays from Medical & Biological Laboratories (Woburn, MA, USA) and from Presage ST2 Assay Critical Diagnostics (San Diego, CA, USA) are commercially available. The latter has received clearance by the U.S. Food and Drug Administration. While the three assays were previously shown to yield comparable results, we cannot entirely exclude the possibility of assay-specific effects [25,34]. The strengths of the study are the very consistent one-to-one matched cohorts, the exactly defined timepoints of blood sampling and the related CTA. Especially, the assessment of AAA progression via CTA at 6-month intervals may represent a more accurate method compared with sonography or larger intervals, as performed in many other countries.

## 4. Materials and Methods

### 4.1. Study Population

**Figure 4:**
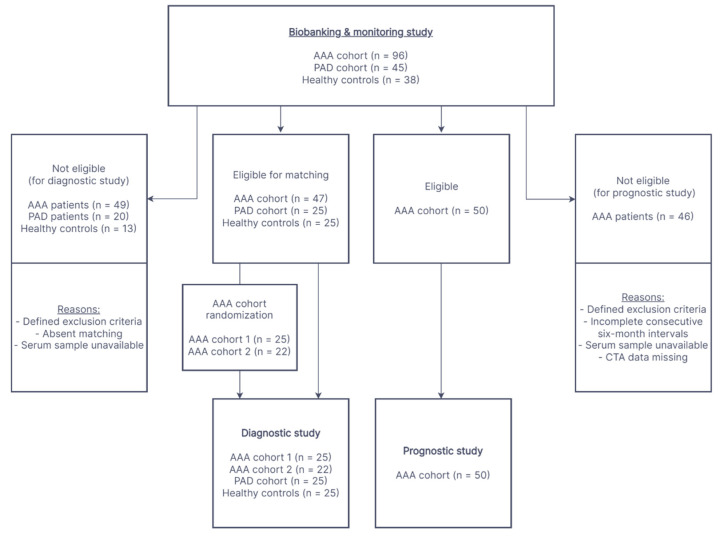
Enrollment flow chart. This flow diagram shows the progress through the phases of participant recruitment for the diagnostic and prognostic study. The cohorts of both study settings were recruited from a biobanking and monitoring study. Abbreviations: AAA, abdominal aortic aneurysm; PAD, peripheral artery disease; CTA, computed tomography angiography.

All studies involving human subjects were conducted in accordance with The Code of Ethics of the World Medical Association (Declaration of Helsinki), and were reviewed and approved by the institutional ethics committee of the Medical University of Vienna (license No. 1729/2014). This study was registered with the Research Registry and the unique identifying number is: researchregistry7647. This single-center retrospective cohort study is reported in line with the STROCSS 2021 and STROBE Guidelines [35,36]. Written informed consent was obtained from healthy controls and all study patients (AAA and PAD patients) who were continuously recruited, diagnosed and monitored at the outpatient clinic at a tertiary university hospital (Division of Vascular Surgery, Department of General Surgery, General Hospital of Vienna, Medical University of Vienna, Vienna, Austria) between 2014 and 2021. To investigate the marker potential of sST2 in a diagnostic setting, a single-center retrospective cohort study was conducted. Patient and control cohorts derived from an underlying biobanking and monitoring study with a total of 96 AAA and 45 PAD patients as well as 38 healthy controls. The study enrollment flow of the participants in the diagnostic study is shown in Figure 4. In the present diagnostic evaluation, data collected from 47 AAA patients (randomly divided into two AAA cohorts: AAA patient cohort 1, *n* = 25 and AAA patient cohort 2, *n* = 22) prior to immanent elective surgical repair via open or endovascular approach were compared with the control groups of 25 PAD patients and 25 healthy controls. The four cohorts were one-to-one matched in age (within 2 years) and sex and as well as possible in smoking habit. Additionally, the diseased cohorts were matched one-to-one with respect to the presence of clinical cardiovascular disease (previous myocardial infarction, angina pectoris, peripheral vascular disease or cerebrovascular disease). The healthy and PAD control groups were continuously recruited from general surgery, urology, angiology and ophthalmology patients (presenting for routine check-ups) with ultrasound-confirmed absence of AAA. In a prognostic setting, an exploratory cohort of 50 AAA patients (also originating from the underlying biobanking and monitoring study) was longitudinally monitored and analyzed over two consecutive 6-month intervals. The study enrollment flow of participants in the prognostic study is shown in Figure 4. In addition, the exclusion criteria for all AAA and PAD patients as well as for healthy controls were recent (<12 months) tumor or chemotherapy, systemic autoimmune or hematological disease, and organ transplantation. Consistently, for individuals in the PAD or healthy control cohorts, any aneurysmal disease of the aorta was an exclusion criterion. No monetary incentivization of study participants for recruitment and retention was provided. The demographics of the study participants were recorded by a structured questionnaire.

### 4.2. Morphometric AAA Analyses, Serum Sample Collection and Determination of sST2 Levels

All study participants underwent peripheral venous blood withdrawal and computed tomography angiography (CTA) imaging at study inclusion and at additionally two longitudinal 6-monthly monitoring visits in the prognostic setting of AAA patients as previously described by our group [9,37]. Individual morphometric AAA analyses were performed with syngo.via (version VB40B, Siemens Healthineers, Forchheim, Germany) and impax EE (Agfa-Gevaert, 17 Mortsel, Belgium) imaging software. Multiple measurements of the same CTA image (maximal AAA diameter) were completed by two trained independent radiology experts, resulting in a mean intra- and interobserver variability range of 0.14 mm and 0.20 mm, respectively [38].

At the same time points, peripheral venous blood samples were collected into serum tubes containing a clotting activator (Greiner Bio One, Kremsmünster, Austria), kept at room temperature for 1 h, centrifuged at 1000× *g* for 10 min and stored in aliquots at −80 °C until analysis. Protein levels of sST2 were quantified using the commercial human ST2/IL-1 R4 DuoSet ELISA kit (R&D Systems, Minneapolis, MN, USA), according to the manufacturer’s instructions and as previously described by our group [39,40].

### 4.3. Statistical Analysis

For describing the patients’ characteristics, the median and interquartile range (IQR) values were calculated for continuous variables, and the absolute and relative frequencies were used for categorical variables. For assessments of statistical significance, non-parametric tests were used (Wilcoxon’s signed rank test for paired samples and the Mann–Whitney U-test for group comparisons of continuous variables as well as Spearman’s test for correlations). For categorical variables, χ^2^ or Fisher’s exact test was applied. Since this was an explorative study, no adjustments for the multiplicity of errors were used. For multiple group comparisons, only significant values were listed in Table 1. The diagnostic marker potential was characterized by receiver operating characteristic (ROC) analysis. Slow versus fast AAA disease progression was defined as a <2 mm or ≥2 mm increase in AAA diameter over the 6 months, reflecting previously published cut-offs in the prognostic setting of 4 mm per year [41]. A specifically tailored log-linear mixed model was used to analyze the association of circulating sST2 protein levels and AAA growth rate in the longitudinal monitoring data (time was incorporated as the main effect and sST2 protein concentrations as the effect modifier), accounting for multiple measurements in patients over time. Further details of the applied log-linear mixed model have been previously described by our group [37,38]. Two-sided p-values below an alpha level of 0.05 were considered to be statistically significant. The data analysis was conducted with SPSS (version 27.0, SPSS Inc., Chicago, IL, USA). The target number of patients per cohort was based on a sample size calculation assuming a 75% difference between the cohorts to be medically significant. In a previous study, the mean sST2 protein level of patients on dual antiplatelet therapy following angioplasty and stenting was 17.47 ng/mL ± 15.33 (standard deviation) [40]. If the true difference between the experimental AAA cohort and respective control cohort means is 13.10 ng/mL, an experimental number of 23 subjects and 23 control subjects would be required. This calculation is based on a probability (power) of 0.80 and a type I error probability (alpha) of 0.05.

## 5. Conclusions

To our knowledge, this is the first study investigating serum sST2 levels in AAA patients addressing their potential association with AAA disease state and progression. Notably, sST2, a well-known biomarker for cardiovascular disease, was shown to be associated with AAA presence but not progression, and could not discriminate AAA from PAD patients. Thus, the results of our analysis may caution the community regarding the limited disease specificity of sST2 as a cardiovascular stress parameter.

## Figures and Tables

**Table 1:** Demographics of AAA patients and control groups in the diagnostic study.

Parameter			Healthy (*n* = 25)	AAA cohort 1 (*n* = 25)	AAA cohort 2 (*n* = 22)	PAD (*n* = 25)	*p*-Value
Metric Variables		*n*	Median (IQR)	Median (IQR)	Median (IQR)	Median (IQR)	
Age (years)		25/25/22/25	67.3 (11)	67.6 (11)	66.3 (10)	68.4 (11)	H:A1—0.009 ^a^
Body mass index (kg/m^2^)		25/24/22/25	27.0 (5.0)	28.1 (6.9)	27.5 (6.3)	25.5 (3.0)	A1:P—0.018 ^a^
Smoking pack-years (py)		17/22/20/23	20.0 (33.0)	45.0 (25.8)	47.5 (23.1)	37.0 (33.0)	H:A2—0.033 ^a^
Mean systolic blood pressure (mmHg)		25/24/19/25	130.0 (15.0)	131.7 (17.0)	130.0 (12.0)	143.0 (33.0)	H:P—0.004 ^a^
Mean diastolic blood pressure (mmHg)		25/24/19/25	80.0 (10.0)	80.0 (12.0)	80.0 (16.0)	71.0 (22.0)	n.s. ^a^
Maximal AAA diameter (mm)		24/20		52.7 (17.5)	48.4 (11.1)		0.546 ^a^
Aortic segment volume (cm^3^)		19/19		114.2 (87.4)	96.3 (83.9)		0.256 ^a^
Maximal ILT diameter (mm)		21/19		13.8 (15.2)	14.5 (11.0)		0.705 ^a^
ILT volume (cm^3^)		19/19		62.9 (80.5)	27.5 (55.9)		0.256 ^a^
Categorical variables			*n* (%)	*n* (%)	*n* (%)	*n* (%)	
Sex	Men		23 (92.0)	23 (92.0)	21 (95.5)	23 (92.0)	0.959 ^b^
	Women		2 (8.0)	2 (8.0)	1 (4.5)	2 (8.0)	
Smoking	Never		8 (32.0)	2 (8.0)	0 (0)	2 (8.0)	H:A1—0.034 ^b^ H:A2—0.010 ^b^ H:P—0.034 ^b^
	Past		13 (52.0)	11 (44.0)	9 (40.9)	15 (60.0)	0.090 ^b^
	Current		4 (16.0)	12 (48.0)	12 (54.5)	8 (32.0)	H:A1—0.015 ^b^ H:A2—0.008 ^b^
AAA family history			2 (8.0)	2 (8.0)	4 (18.2)	2 (8.0)	0.352 ^b^
Hypertension			13 (52.0)	24 (96.0)	16 (72.7)	23 (92.0)	H:A1—<0.001 ^b^ H:P—0.002 ^b^ A1:A2—0.040 ^c^
Hyperlipidemia			7 (28.0)	21 (84.0)	17 (77.3)	19 (76.0)	H:A1—<0.001 ^b^ H:A2—0.001 ^b^ H:P—0.001 ^b^
Coronary heart disease			0 (0)	11 (44.0)	8 (36.4)	9 (36.0)	H:A1—<0.001 ^b^ H:A2—0.001 ^c^ H:P—0.002 ^c^
Myocardial infarction			0 (0)	6 (24.0)	5 (22.7)	4 (16.0)	0.125 ^b^
Stroke			1 (4.0)	3 (12.0)	1 (4.5)	5 (20.0)	0.214 ^b^
Diabetes mellitus type 2			3 (12.0)	13 (52.0)	3 (13.6)	9 (36.0)	H:A1—0.002 ^b^ H:P—0.047 ^b^ A1:A2—0.006 ^b^
COPD			1 (4.0)	4 (16.0)	11 (50.0)	5 (20.0)	H:A2—<0.001 ^b^ A1:A2—0.013 ^b^ A2:P—0.030 ^b^
Antiplatelet therapy			5 (20.0)	22 (88.0)	18 (81.8)	21 (84.0)	H:A1—<0.001 ^b^ H:A2—<0.001 ^b^ H:P—<0.001 ^b^
Anticoagulation therapy			2 (8.0)	3 (12.0)	4 (18.2)	4 (16.0)	0.741 ^b^
Antihypertensive therapy			13 (52.0)	24 (96.0)	19 (86.4)	20 (80.0)	H:A1—<0.001 ^b^ H:A2—0.012 ^b^ HC:P—0.037 ^b^
Lipid-lowering agents			4 (16.0)	23 (92.0)	17 (77.3)	23 (92.0)	H:A1—<0.001 ^b^ H:A2—<0.001 ^b^ H:P—<0.001 ^b^

Abbreviations: *n*, number of individuals; AAA, abdominal aortic aneurysm; PAD, peripheral artery disease; IQR, interquartile range; ILT, intraluminal thrombus; COPD, chronic obstructive pulmonary disease; n.s., not significant; H, healthy control cohort; A1, AAA patient cohort 1; A2, AAA patient cohort 2; P, PAD patient cohort. Values are given as medians with the IQR for continuous variables or n (%) for categorical variables. The following statistical tests were applied: ^a^ Wilcoxon’s signed rank test; ^b^ χ^2^ test; ^c^ Fisher’s exact test.

**Table 2:** Demographics of AAA patients in the prognostic study.

Parameter			AAA cohort (*n* = 50)
Metric Variables		*n*	Median (IQR)
Age (years) at first visit		50	71.4 (12.0)
Body mass index (kg/m^2^)		50	27.6 (6.0)
Smoking pack-years (py)		46	40.0 (28.5)
Mean systolic blood pressure (mmHg)		50	132.7 (20.0)
Mean diastolic blood pressure (mmHg)		50	79.2 (11.0)
Maximal AAA diameter (mm) at first visit		50	43.7 (11.6)
Aortic segment volume (cm^3^) at first visit		45	68.2 (64.2)
Maximal ILT diameter (mm) at first visit		46	12.3 (11.8)
ILT volume (cm^3^) at first visit		45	24.5 (40.8)
Categorical variables			*n* (%)
Sex	Women		7 (14.0)
	Men		43 (86.0)
Smoking	Never		4 (8.0)
	Past		23 (46.0)
	Current		23 (46.0)
AAA family history			8 (16.0)
Hypertension			45 (90.0)
Hyperlipidemia			42 (84.0)
Coronary heart disease			21 (42.0)
Myocardial infarction			9 (18.0)
Stroke			5 (10.0)
Diabetes mellitus type 2			15 (30.0)
COPD			10 (20.0)
Antiplatelet therapy			46 (92.0)
Anticoagulation therapy			8 (16.0)
Antihypertensive therapy			44 (88.0)
Lipid-lowering agents			47 (94.0)

Abbreviations: *n*, number of individuals; AAA, abdominal aortic aneurysm; IQR, interquartile range; ILT, intraluminal thrombus; COPD, chronic obstructive pulmonary disease. Values are given as the median with IQR for continuous variables or *n* (%) for categorical variables.

## Data Availability

The datasets used and/or analyzed during the current study are available from the corresponding author on reasonable request. This study was registered with the Research Registry and the unique identifying number is: researchregistry7647. This hyperlink can be used to publicly access our specific registration: https://www.researchregistry.com/browse-the-registry#home/registrationdetails/620cd5f5137159001eeec81c/, accessed on 6 August 2022.
